# Real-Time Rain Rate Evaluation via Satellite Downlink Signal Attenuation Measurement

**DOI:** 10.3390/s17081864

**Published:** 2017-08-12

**Authors:** Filippo Giannetti, Ruggero Reggiannini, Marco Moretti, Elisa Adirosi, Luca Baldini, Luca Facheris, Andrea Antonini, Samantha Melani, Giacomo Bacci, Antonio Petrolino, Attilio Vaccaro

**Affiliations:** 1 Dipartimento di Ingegneria dell’Informazione, University of Pisa, Pisa 56122, Italy; ruggero.reggiannini@iet.unipi.it (R.R.); marco.moretti@iet.unipi.it (M.M.); 2 CNIT-Laboratorio Nazionale di Radar e Sistemi di Sorveglianza, Pisa 56124, Italy; elisa.adirosi@artov.isac.cnr.it (E.A.); luca.facheris@unifi.it (L.F.); 3 Institute of Atmospheric Sciences and Climate, CNR, Rome 00133, Italy; l.baldini@isac.cnr.it; 4 LaMMA Consortium, via Madonna del Piano 10, Sesto Fiorentino (FI) 50019, Italy; antonini@lamma.rete.toscana.it; 5 CNR IBIMET, Via Giovanni Caproni 8, Firenze 50145, Italy; s.melani@ibimet.cnr.it; 6 MBI Srl, Pisa 56121, Italy; gbacci@mbigroup.it (G.B.); apetrolino@mbigroup.it (A.P.); avaccaro@mbigroup.it (A.V.)

**Keywords:** satellite communication, rain fading, rain attenuation, meteorology, electromagnetic propagation in absorbing media, microwave propagation, weather sensors

## Abstract

We present the NEFOCAST project (named by the contraction of “Nefele”, which is the Italian spelling for the mythological cloud nymph Nephele, and “forecast”), funded by the Tuscany Region, about the feasibility of a system for the detection and monitoring of precipitation fields over the regional territory based on the use of a widespread network of new-generation Eutelsat “SmartLNB” (smart low-noise block converter) domestic terminals. Though primarily intended for interactive satellite services, these devices can also be used as weather sensors, as they have the capability of measuring the rain-induced attenuation incurred by the downlink signal and relaying it on an auxiliary return channel. We illustrate the NEFOCAST system architecture, consisting of the network of ground sensor terminals, the space segment, and the service center, which has the task of processing the information relayed by the terminals for generating rain field maps. We discuss a few methods that allow the conversion of a rain attenuation measurement into an instantaneous rainfall rate. Specifically, we discuss an exponential model relating the specific rain attenuation to the rainfall rate, whose coefficients were obtained from extensive experimental data. The above model permits the inferring of the rainfall rate from the total signal attenuation provided by the SmartLNB and from the link geometry knowledge. Some preliminary results obtained from a SmartLNB installed in Pisa are presented and compared with the output of a conventional tipping bucket rain gauge. It is shown that the NEFOCAST sensor is able to track the fast-varying rainfall rate accurately with no delay, as opposed to a conventional gauge.

## 1. Introduction

Rainfall is a critical factor of water cycle dynamics as it is a vector for energy exchange between atmosphere, sea and land. Moreover, it has a key role for water supply; rainfall monitoring is an important tool to be used for water management, especially in areas characterized by water scarcity. Some other important practical applications also concern agriculture and human welfare. Finally, the measurement, the monitoring and the forecasting of rainfall are different phases of meteorological studies substantial for the management of alerts and the assessment of environmental risks due to floods and landslides. They also play a key role in climatological studies, as the rainfall amount is an important climate index. The knowledge of the instantaneous rainfall intensity during precipitation events is very useful in urban areas as it allows a better understanding of hydrological dynamics for the correct interpretation of the complex infiltration and runoff processes. Urban hydrological flows are constrained by the drainage system and highly dependent on the rainfall intensity. In order to produce current estimates of the system conditions, the real-time rainfall data are very useful; they are also able to highlight short and very intense events.

Several methods for measuring precipitation exist, based on different physical principles, direct and indirect methods, and ground and space-based systems. A complete up-to-date review regarding the fundamental and technical aspects of rainfall estimation is offered by [1], together with the description of the physical operating principle, an overview of the scientific knowledge and the description of existing methods and future emerging technologies. Although indirect methods based on remote measurements (weather radars and weather satellites) are widespread, they unfortunately do not allow the providing of a high accuracy of measurements while providing a correct view of the spatial distribution of precipitation. Conversely, point observations (rain gauges and disdrometers) provide the most direct measurements of precipitation, but they are referred only to the specific point of installation and in most cases (rain gauges) always produce time-accumulated values. The use of point gauges for monitoring large areas requires the deployment of a large number of sensors, leading to a cost rise.

The combination of remote sensing and in situ observations for precipitation estimation is particularly suitable for now-casting purposes [2], as the indirect measurements of radar and satellites are related to the presence and vertical development of clouds, atmosphere microphysics as well as the precipitation; this information is very useful to interpret the weather phenomena properly.

### 1.1. Ground-Based Instruments

Several ground-based instruments exist [3] which take advantage of different rainfall characteristics and various physical principles of measurements. There are some systems that directly measure the amount of rainfall by observing the increase in the volume and the weight of the water in a collection container, using mechanisms based on float (float gauges), springs or system balance (weigh gauges) or mechanical parts driven by a known amount of water (tipping bucket gauges). Other types of systems are the disdrometers that measure the drop size distribution and, in some cases, also the terminal velocity of falling hydrometeors, and can help to distinguish different types of precipitation as rain, graupel, hail or snow. Various types of technologies are employed for implementing disdrometers systems [4]. The kinetic energy of falling drops can be measured by means of acoustic or displacement methods. Optical disdrometers employ imaging (2–3D video disdrometers) or laser technologies to measure raindrop microstructure by means of non-catching methods, reducing the effects of splashing and drops break up during the measurement process. Finally, the use of active microwave sensors pointing in the vertical direction constitutes an additional type of disdrometer that exploits Doppler power spectra of power received from falling drops.

Regarding the types of error sources, the main problem for point measurements is the difficulty of extrapolating the representativeness to an area, due to the high spatial variability of rainfall. During the measurement process, both systematic and random errors can occur. Errors due to the effect of wind, the evapotranspiration losses, the site-dependent instrument exposure, and the incorrect calibration fall within the first type. Malfunctioning problems, instrumental errors and observational effects can vary depending on the specific case, and therefore produce random effects. Some solutions exist for reducing the errors of the measuring instrument and the correction accuracy can be dependent on the observed phenomena [3]. Tipping bucket rain gauges (TBRs) are widely used, due to their simplicity and reliability, which reduces installation and maintenance costs. Several networks have been deployed around the globe for monitoring rainfall for a series of applications spanning from hydrogeological vigilance to agronomic management practices and to provide ground validation products for radar and satellites rainfall estimation systems.

An in-depth literature review of the issues and problems pertaining to the use of TBRs, featuring laboratory testing and field evaluation, is reported in [5]. Tipping-bucket data are rather inaccurate and their readings require thus some adjustment [6] or cumbersome correction procedures [7]. The accuracy of some types of TBRs is experimentally evaluated in [8] and [9].

The major problems of the TBRs are that they cannot measure instantaneous rainfall, but provide, at discrete-time instants, measurements of the water quantity relative to a time interval. For this reason, the information about rainfall spikes and peaks, essential in hydrological applications to solve water balance, is not given in the raw measurements. This is particularly critical, especially in very small catchment areas, such as urban ones, which are vulnerable from the hydrological point of view, as the hydrological processes develop very fast. In order to have continuous-time rain-rate estimates, algorithms must be applied to the measured data for the reconstruction of an instantaneous rain rate from the tipping bucket measurements. The simplest way is a linear interpolation based on the ratio between the bucket size and the time intervals between two tips. Unfortunately, this method only provides an averaged value and cannot reconstruct the instants of zero rain. In [10], a cubic spline, constructed of third-order polynomials, is used to interpolate discrete TBR tip records into one-minute rain rates during a rain event.

A survey on the state-of-the-art rainfall intensity gauges, with the relevant physical principles, is presented in Section 2.2 of [11]. The response time of an instrument plays an important role in assessing its suitability for rainfall intensity measurements. The dynamic response of conventional rainfall intensity gauges is shown in Figure 23 of [11], which shows that the response time and/or time delay with respect to the applied intensity step can vary from 1 up to 5 min. Also from Figure 23, it can be noted that the step response (and also the accuracy of the measurement) is strongly dependent on the intensity, with a more apparent delay effect when operating in the presence of medium-to-low rainfall intensities.

### 1.2. Weather Radars

Weather radars are used for monitoring precipitation by most weather services. They are able to detect, quantify and monitor the evolution of precipitation events over a wide area (circular area with a radius ranging from approximately 70 to 200 km, depending on the frequency used) with a high spatial (less than 1 km, although not constant) and temporal (5–10 min) resolution. Furthermore, they can provide a 3D representation of the precipitation event, therefore allowing us to get a deep insight into the characteristics of rainfall and in general of the ongoing microphysical processes governing precipitation. Typically, weather services use radars by arranging them in a networked configuration, in order to obtain seamless coverage of nation-wide areas for meteorological and hydrological applications. However, weather radars perform indirect measurements of rainfall, obtaining precipitation estimates by converting radar measurements into rainfall rate using appropriate conversion algorithms [12]. In the most common configuration, weather radar radiates a train of short duration electromagnetic pulses that, in the presence of precipitation within the radar volume defined by the antenna pattern, is scattered in all directions. The wave’s energy backscattered toward the radar receiver (weighted by the inverse of squared distance) is nearly proportional to a weather radar parameter called the radar reflectivity factor (*Z*), which depends on the number and size distribution of hydrometeors and their absorption and scattering properties related to the density and shape of the hydrometeors, radar wavelength and temperature. Most of the operational weather radars have adopted the dual polarization technology. Such radars are capable of transmitting pulses on both horizontal and vertical planes (following alternate or simultaneous schemes), providing additional information related to the shape and size of the hydrometeors, which can be used to improve the accuracy of the rainfall estimation algorithm and to obtain information on the precipitation microphysics [13,14]. Quantitative precipitation estimation (QPE) by weather radar can be affected by many sources of errors (see [15,16] and the references therein), that can be related to (i) errors in radar measurements, such as radar miscalibration, presence of ground clutter, partial beam blocking, propagation effects and precipitation attenuation effects; (ii) errors in the conversion of radar measurements into rainfall rate at ground, due to the vertical variability of the precipitation system and to the assumption made about the drop size distribution (DSD, namely the number of drops per unit of drop diameter interval and per unit volume) used to obtain retrieval algorithms, and (iii) errors that increase with the distance from the radar antenna, such as the broadening of the radar beam (which causes an increase of the radar sampling volume with range), and the time-height ambiguity between the rainfall rate obtained from radar measurement at certain height (that increases with the distance) and the rainfall rate at the ground.

### 1.3. Microwave Links

Recently, the use of microwave (MW) links has been introduced in several studies for the operational estimation of precipitation from tropospheric radio links [17]. Received signal levels of MW links provide complementary information with respect to rain gauges, disdrometers, radars and satellites. An important advantage of using such links is the cost reduction achieved by exploiting already existing tools developed for other purposes. Concerning satellite links, studies of attenuation due to clouds and precipitation along the propagation path have been intensively carried out, mainly in order to optimally design satellite communication systems. The idea of using such a “signal of opportunity” for measuring precipitation is more recent. We mention hereafter some promising results provided by experiments which compare rain rate measurements by rain gauge networks or weather radars with rain estimates obtained from the evaluation of the attenuation in commercial satellite links at Ka [18] or Ku band [19], and also by experiments aiming at retrieving rainfall fields from geostationary broadcast television satellite links [20,21]. In this paper, we show an innovative application based on acquiring measurements of satellite downlink signal attenuation measurements for estimating rainfall fields. The algorithm used for retrieving rainfall is described, together with the architecture of a first prototype of the measurement system. A TBR sensor has been installed for comparison and validation purposes. Some preliminary results of rainfall retrieval are illustrated and discussed for some selected case studies.

## 2. Architecture of the Real-Time Wide-Area Rain-Rate Measurement System

### 2.1. Rationale and Underlying Idea

NEFOCAST (for the sake of simplicity, the more concise name NEFOCAST, originating from the contraction of “Nefele”, which is the Italian spelling for the mythological cloud nymph Nephele, and “forecast” will be used hereafter in place of the, more involved, official name of the project (SVI.I.C.T.PRECIP.) is a research project funded by the government of the Tuscany Region (Italy). Started in September 2016 for a duration of 24 months, NEFOCAST is based on an innovative solution: It uses a dense population of interactive satellite terminals (ISTs) that serve as both weather sensors and modems to send the measurements to a dedicated now-casting platform to receive back rainfall information and potential alerts. The IST used for the project is named SmartLNB (smart low-noise block converter) and is an innovative product, introduced by the satellite operator EUTELSAT with the ambition to replace the traditional direct-to-home (DTH) receivers, providing at the same time additional interactive services through a narrowband return channel. The SmartLNB is a new-generation electronic feed connected to an antenna with an embedded transmitter for interactive applications. Such terminals serve as both weather sensors and modems to send the measurements to a dedicated center (the NEFOCAST service center) that generates precipitation products and eventually related weather warnings. Since ISTs are low-cost and low-power RF transceivers that are also equipped with a traditional satellite television (TV) decoder, they can replace the current population of DTH receivers. The NEFOCAST precipitation maps will be therefore based on capillary measurements, constantly and rapidly updated. Such system presents interesting aspects when compared to other consolidated precipitation measurement instruments.

Due to its promising results, the NEFOCAST project received the endorsement from EUTELSAT, a leading satellite operator, and Météo France, a big player in the weather forecast market, which are both interested in setting up a collaboration based on the project results to develop new services. The business related to weather data has undergone an exponential growth in the last few years and, accordingly, provider companies of weather data grew very fast in terms of value and proposition.

### 2.2. System Architecture and Description

**Figure 1:**
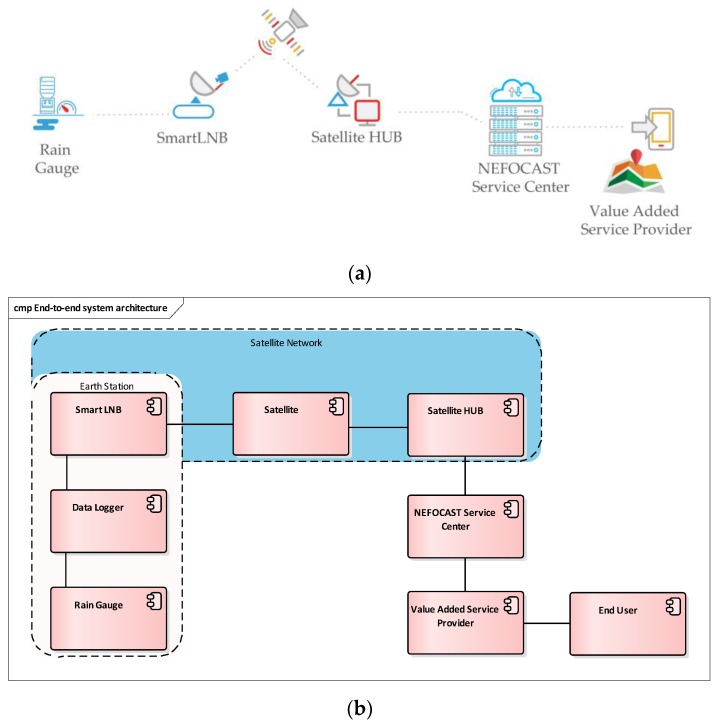
(**a**) NEFOCAST’s experimentation network for real-time wide-area rain-rate measurement and (**b**) its high-level end-to-end architectural description.

Figure 1 illustrates NEFOCAST’s experimentation network for real-time wide-area rain-rate measurement (Figure 1a). Its relevant high level end-to-end architectural description is shown in Figure 1b. The experimentation network consists of a number of Earth stations, wherein each station is equipped with a SmartLNB device (see Section 2.1) interconnected to the satellite network. Some of the installations include also a TBR with a data logger, for validation purposes.

The NEFOCAST Service Center collects information from the satellite network and the rain gauges, generating rain field maps that are then shared with a number of value added service providers (VASPs) that use them to offer dedicated services to end users.

### 2.3. SmartLNB and Satellite Network

The SmartLNB [22] is an innovative IST engineered for Internet of Things (IoT) and machine-to-machine (M2M) applications (and hence it is a “smart” low-noise block converter). It is a bi-directional communication system which makes use of the digital video broadcasting satellite second generation (DVB-S2) [23] on the forward link (from the satellite hub to the SmartLNB) and fixed-satellite interactive multimedia (F-SIM) [24] on the return link (from the SmartLNB to the satellite hub) communication protocols.

**Figure 2:**
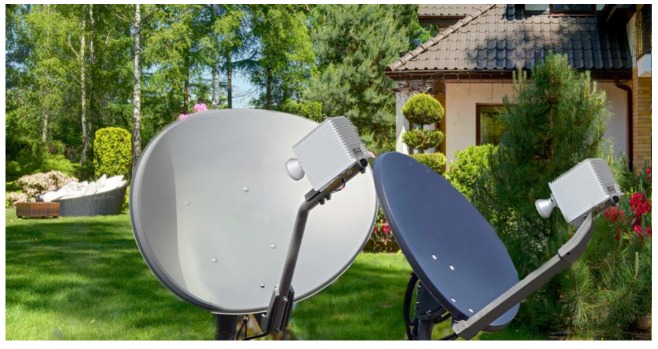
Smart low-noise block converter (SmartLNB).

The unit, composed of an indoor unit (IDU) and an outdoor unit (ODU), is formed by a 70 cm-diameter satellite dish and the actual SmartLNB appliance (see Figure 2).

**Figure 3:**
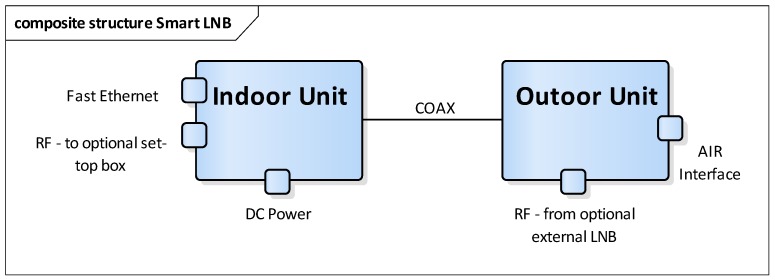
Architecture of the SmartLNB.

The IDU will provide electrical power to the whole system, and can be interconnected to a local network to exchange data via satellite and optionally to a satellite set-top box to receive standard broadcast channels. The ODU is interconnected to the IDU using a coaxial cable. It has an optional RF input for an external LNB, to be used in conjunction with another existing satellite receiver (Figure 3).

The SmartLNBs used for the experimentation operate in Ku-band for both the forward and return links. The quality of these links is affected by weather conditions: Rain rate and SmartLNB signal attenuation (both transmitted and received) are highly correlated. The algorithms studied in the project allow the translation of signal attenuation measurements into rain rate estimates, which means that the SmartLNB can be used also as a weather sensor.

In fact, it can measure the power of the received DVB-S2 signal and the associated signal-to-noise ratio with a resolution of 0.1 dB in real time. Regarding the network, the hub is able to measure the power level and the signal-to-noise ratio of the F-SIM traffic sent by all the SmartLNBs composing the network. Both measurements are stored in the hub database and accessed by the NEFOCAST Service Center for further processing.

At any time, the satellite hub stores information on the signal attenuation on both forward and return links for all the SmartLNBs. Forward link attenuation is measured by the SmartLNBs and transmitted to the hub, while return link attenuation is directly measured by the hub subsystems.

The hub database also stores geographic coordinates of all the SmartLNBs in the network, which are provided during their installation and commissioning phase.

The SmartLNB is able to send weather information with a very dense granularity in the time domain (e.g., sub-second measurements) and to receive feedbacks simultaneously from the NEFOCAST service center, such as weather alerts. This significantly simplifies and optimizes the now-casting network architecture, as the SmartLNBs can simultaneously serve as both dense and scalable weather sensors, and communication nodes.

The network of Smart LNBs deployed for the experimentation serve as both weather sensors and modems to send the rain gauge measurements to the NEFOCAST service center.

### 2.4. Tipping Bucket Rain Gauges Sensors

**Figure 4:**
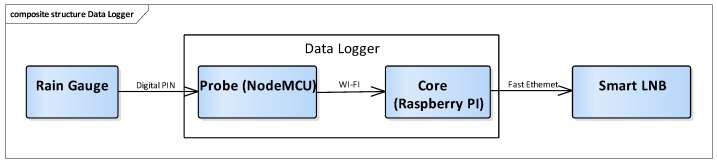
Architecture of the Earth station rain gauge sensor.

After a pre-set amount of precipitation fall, the rain gauge lever tips and sends an electrical signal to the data logger. Periodically, the data logger sends the log of the received data to the NEFOCAST service center using the satellite network. Figure 4 shows the architecture of the rain gauge sensor system in the Earth station.

For each tip, the rain gauge collects an amount of water equivalent to 0.2 mm rainfall. Tips are generated by a reed switch within the rain gauge. The data logger keeps track of each tip: These data are then integrated over a configurable time interval in the NEFOCAST Service Center. Debouncing is applied to the rain gauge signal in order to avoid possible false detections. The data logger is divided in two parts: A probe and a core module. The probe is installed inside the rain gauge and communicates with the core module using a dedicated Wi-Fi link. This probe is implemented using a NodeMCU ESP8266 microcontroller. Signals from the rain gauge are read on one of the probe’s general purpose input output (GPIO) pins and a multicast user datagram protocol (UDP) message is generated for each of them. Each data logger has an associated unique ID that is transmitted within the messages.

This choice allows the use of the same data logger to collect statistics from multiple probes at the same time. The core module listens for multicast UDP packets coming from the probes, aggregates and forwards them to the NEFOCAST service center using the satellite link provided by the SmartLNB. Messages are also stored within the data logger: In the case of communication issues with the NEFOCAST service center (e.g. no satellite link available due to heavy rain), messages that have not been yet delivered will be sent as soon as the connection will be restored.

### 2.5. NEFOCAST’s Space Segment

The NEFOCAST system uses a bi-directional geostationary satellite network. The link connecting the ISTs to the gateway is used by the ISTs to access the terrestrial infrastructure via the satellite. The forward link transports the traffic generated by remote servers on earth and addressed to the ISTs via the satellite. EUTELSAT 10A (EUT 10A) geostationary satellite is used by NEFOCAST SmartLNBs for both the return and the forward links. The satellite is located at ten degrees east longitude and provides Ku-band widebeam footprints serving Europe. Table 1 describes the characteristics of the space segment.

### 2.6. NEFOCAST's Service Center

Measurements of SmartLNB power levels on both the forward and the return links are periodically collected by the NEFOCAST service center, which interacts with the satellite hub management system using a dedicated application programming interface (API). These data are processed to get the signal attenuation on the path between each SmartLNB and the satellite, which will be then translated into rain rate estimates. The NEFOCAST Service Center also stores data coming from the rain gauges of its network. It is possible to compare rain rates observed by the Smart LNBs with the values measured by the rain gauges.

Data coming from the sensor network are further processed using a specific algorithm that generates real time rain field maps. These output data (both precipitation maps and rainfall estimated by ground stations) will be available to service integrators through a public API. It will then be possible to show them in end-user applications or integrate them in other more complex systems (e.g., weather forecast, logistics, business strategy engines, etc.). In both cases, these activities are developed by VASPs. In the context of the experimentation an existing alerting application, developed by one of the project partners, has been selected to be used for data presentation and alerting.

### 2.7. Market Opportunities

The technologies developed within the project will be used to obtain very detailed estimates of precipitation. The NEFOCAST Service Center is able to provide real time precipitation data for now-casting applications, since it is primarily thought of as a tool for monitoring and detect precipitating events, even severe events.

In particular, it will be possible to obtain information with a temporal resolution much higher than what provided by other monitoring systems. These results will be valuable and significant for the prevention of natural disasters and for the continuous monitoring of basins and their precipitation status, as well as for other applications in the productive and civil fields. A considerable number of companies provide weather data in XML or CSV format at very convenient prices. Compared to existing market solutions, the main advantages of the NEFOCAST solution are:

- Higher accuracy, frequency and detail for the provided information, especially when combined with other forecast systems;
- The availability of all information in one single data center;
- The possibility to serve a large number of professional users using the same technological platform.

The NEFOCAST solution could provide interesting perspectives in reducing costs and having a high quality of the provided data at the same time. The prospective is to expand the covered area from one single country in one year after the project’s end to ten European countries in the forthcoming five years. The higher the number of installed SmartLNBs, the more accurate and detailed the precipitation estimation is. A very dense network of sensors, such as the one that could be available using home satellite receivers, could lead to a downscaling of the field’s coverage. Such a resolution increase will be beneficial when dealing with extreme events such as sudden flooding in urban areas to mitigate the exposure to risk for the population.

## 3. Earth-Space Radio Links in the Presence of Rain

### 3.1. Effects of Rain on Earth-Space Radio Links

**Figure 5:**
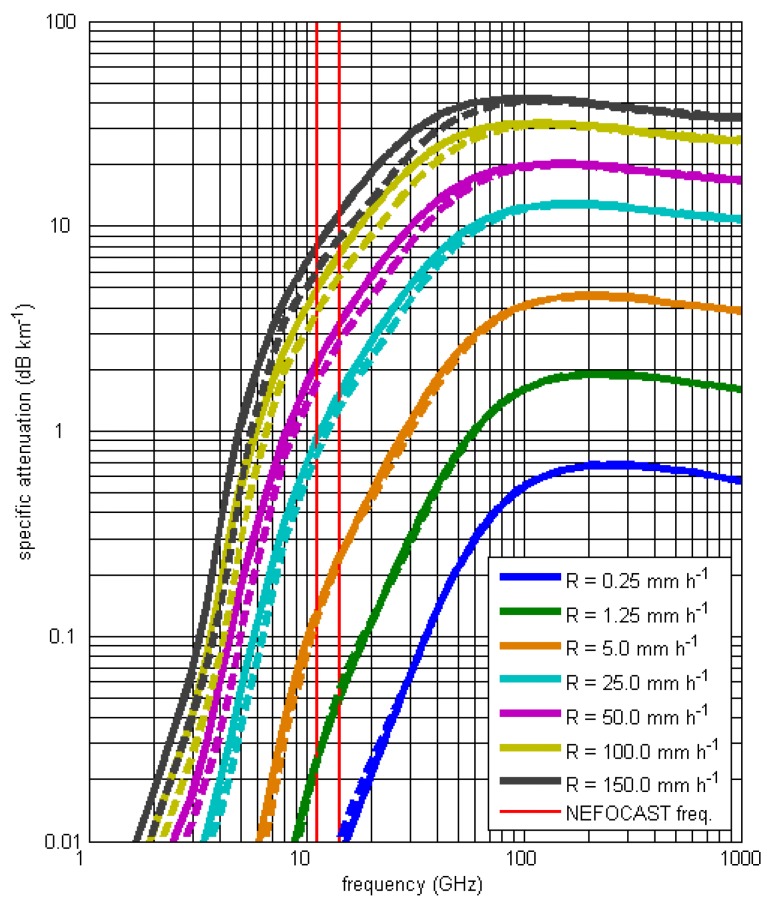
Specific attenuation in dB/km vs. carrier frequency in GHz (from recommendation ITU-R P.838-3 [29]), with the rain rate (mm/h) as the parameter. Solid lines and dashed lines refer to horizontal and vertical polarization, respectively.

At frequencies higher than 5 GHz (as those considered in the NEFOCAST project), the electromagnetic wave that propagates from satellite to the Earth interacts with atmospheric gases and hydrometeors [25]. It has been shown that the attenuation due to the presence of liquid hydrometeors is the predominant mechanism that produces the degradation of the signal [26]. However, the effects of hydrometeors in the mixing phase (such as wet snow, graupel and melting hydrometeors) cannot be neglected. The interaction between electromagnetic waves and rain produces an attenuation of the propagating signal due to the combination of two mechanisms: Scattering in all the directions and absorption, causing a total, path-cumulated attenuation of the signal reaching the receiver. The absorption and scattering produced by liquid particles (such as raindrops) depends on the frequency and the polarization state of the incident wave, and on the characteristics of the precipitation; namely, the size and shape of the raindrops. Different theories have been developed to model the scattering phenomena by raindrops. The theory of Rayleigh–Gans scattering is applicable to small (*D* << *λ*, where *D* is the diameter of the drops), non-absorbing and spherical drops, while the Mie theory is applicable to spherical raindrops with *D* ≅ *λ*. Finally, the scattering amplitudes of non-spherical drops cannot be computed analytically. However, several numerical techniques are available for calculating the scattering properties, such as the T-matrix method [27]. Therefore, the size distribution of particles plays a central role in the estimation of the total attenuation undergone by the signal that propagates along the radio link and due to rain. The DSD can be modelled by different theoretical parametrizations (see [28] and the references therein) or can be measured by disdrometers. Knowing the DSD, either measured or simulated, the specific attenuation *k* (dB/km) due to the presence of precipitation can be obtained as

(1) $$k=4.343\times 10^{-3}\;\int_{0}^{\infty }\sigma_{E}\left(q,D,\lambda ,T,h\right)\;N\left(D\right)\;dD,$$

where *σ_E_* is the extinction cross section (namely, the area that when multiplied by the power density of incident wave, gives the power loss due to absorption and scattering) that depends on the polarization (*q* is a label that stands for “vertical”, “horizontal”, or “circular”), wavelength (*λ*) of the incident waves, diameter (*D*), type of the particles (*h* is a label that stands for “rain drop”, “hail”, “snow flake”, “graupel”, or “particle in a mixing phase”) and on the temperature (*T*), while *N*(*D*) is the DSD. The ITU-R P.838-3 recommendation [29] provides, for frequencies ranging from 1 to 1000 GHz, the coefficients of the relation *k* vs. rain rate *R* given in the form of an approximate power law, for horizontal and vertical polarizations. Figure 5 shows clearly that, for the operational frequencies adopted in the NEFOCAST project, attenuation due to severe precipitation (*R* > 100 mm/h) ranges from 3.6 to 11.3 dB/km, depending on frequency and polarization. Similar results can be obtained considering measured DSDs (see Section 4.4). The advantage of using measured DSDs is that, from a climatological point of view, they are more representative of the precipitation characteristics of the geographical area where they have been sampled; however, they can be affected by measurement errors. In addition, it is possible to characterize the error due to the approximation of the *R*-*k* relation with respect to the variability of the DSD, which is not known from the ITU-R P.838-3 recommendation [29].

**Figure 6:**
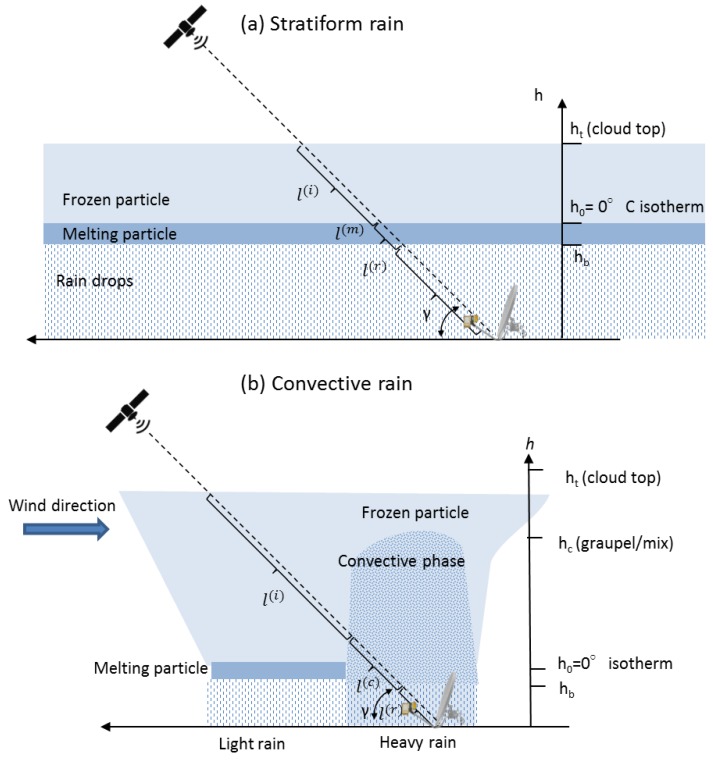
Geometry of an Earth-space radio link in two different meteorological conditions: (**a**) stratiform precipitation and (**b**) convective precipitation.

Figure 6 shows the geometry of the Earth-space radio-link adopted during the NEFOCAST project, where *γ* is the elevation angle (about 40° in this case). The smart-LNB provides the total attenuation (in dB) along the radio path between satellite and the ground. As mentioned before, the attenuation due to frozen hydrometeors can be neglected, and the majority of the attenuation is due to rain (namely along the path called *l*^(*r*)^ in Figure 6). During operations, two different reference precipitation regimes may show up, namely, convective rain and stratiform rain, which are associated with different environmental conditions and precipitation growth mechanisms. Several different definitions of stratiform and convective rain can be found in the literature. However, for the scope of the present study, we can say that convective rain is associated with the presence of vertical wind (up or down-draft) and high rainfall rates. Furthermore, it usually covers a small area and has a huge vertical extension, without a well-defined separation layer between frozen and liquid hydrometeors as sketched in Figure 6b. Instead, in stratiform precipitation, the vertical wind is almost absent, the precipitation covers an extended area and is fairly homogeneous on the horizontal level, as shown in Figure 6a. Finally, along the vertical, three different layers characterized by the presence of different types of hydrometeors are typically well identifiable. At the cloud top, frozen particles are present that start to melt when the environmental temperature approaches 0 °C. The melting layer has a thickness of few hundreds of meters [30], and below its bottom border there are hydrometeors in liquid phase.

### 3.2. Conventional Statistical Models for Attenuation Prediction in Earth-Space Radio Links in the Presence of Rain

The two most used statistical models [31] for the prediction of the attenuation affecting a satellite link are the ITU-R (see recommendation ITU-R P.618-11 [32]) and the Crane Global [33] models. Furthermore, the technical literature presents a plenty of models that exhibit good prediction accuracy performance, such as those introduced in [34,35,36].

For any geographical location, as a starting point, all the models take the statistical information about the local precipitation expressed as the complementary cumulative distribution function (CCDF) of the rain rate R as *F_R_*(*x*) = Pr(*R* > *x*) (see e.g., recommendation ITU-R P.837-6 [37]). This information is then analytically used for a prevision of the statistics of the signal attenuation *A* (dB) that will be experienced on the satellite link. Summarizing, both models yield a relationship Φ between a given threshold value $\bar{R}$ of the rain rate R, together with the associated probability *p* = *F_R_*($\bar{R}$) = Pr(R > $\bar{R}$), and a threshold value $\bar{A}$ of the signal attenuation *A* having the same probability of being exceeded, i.e.,

(2) $$\mathrm{given}\;\bar{R}:p=F_{R}(\bar{R})=\mathrm{Pr}(R>\bar{R}),\;\mathrm{the}\;\mathrm{model}\;\mathrm{yields}\;\bar{A}=\Phi (\bar{R},p):p=F_{A}(\bar{A})=\mathrm{Pr}(A>\bar{A}).$$

## 4. Direct Method for the Estimation of the Rain Rate

### 4.1. Limitations of the Statistical Models

Though adequate for the prediction of the rain fading attenuation when dimensioning an operational margin of a satellite link, the above models definitely reveal themselves as not suitable for the inverse task; i.e., the derivation of an accurate rain rate estimate starting from signal attenuation measurements. Actually, customary statistical models only yield a relationship between the CCDF of the rain rate and that of the signal attenuation, i.e., $\mathrm{Pr}(R>\bar{R})=p\to \mathrm{Pr}(A>\bar{A})=p$, while the relationship Ψ(⋅) needed for the proposed NEFOCAST rain rate sensing system should be of the type $\hat{R}=\Psi (\hat{A})$, where $\hat{A}$ is a measured attenuation and $\hat{R}$ is the corresponding estimated rain rate.

### 4.2. Direct Model Concept

By the “direct method” we mean a direct mapping rule Ψ(⋅) from a signal attenuation measure $\hat{A}$ to an estimate $\hat{R}$ of actual rainfall rate, without the need to resort to intermediate or auxiliary (location dependent) statistical descriptions, such as the probabilities $F_{A}(\bar{A})$ and $F_{R}(\bar{R})$ involved in statistical models (see Section 3.2). In comparison with the above-described (inverse) ITU-R and Global Crane approaches, a direct method is expected to provide better accuracy in estimating the rainfall rate, since it utilizes the actual knowledge of a larger number of parameters affecting path attenuation; notably, the height of the liquid precipitation layer.

### 4.3. Rain Height

Rain height is a key parameter for both direct and statistical models. Through weather radar data, one can estimate the actual height and width of the melting layer. Different signatures of radar measurements can be used, depending on the available measures and the observation geometry. The most known signature is the “bright band”, a layer of enhanced reflectivity below the 0 °C isotherm that is due to large and wet snowflakes that melt during their fall. This signature can be observed both at vertical incidence and in the quasi-horizontal scan performed by operational radar systems, where it appears as a circular band with greater reflectivity. The Doppler velocity signature reflects the increase of velocity that takes place during melting: In vertically pointing measurements, Doppler velocity exhibits a sudden increase that ends where melting of particles is complete [30]. Therefore, the position where such enhanced reflectivity ends indicates the height where melting is complete. Such a feature is observable only at vertical incidence. Dual polarization measurements present specific signatures of the melting layer, the most evident being the dip in the copolar correlation coefficient, which is determined by the heterogeneity of scatterers (iced, liquid or partially melted) within a radar resolution volume during the melting process. In quasi-horizontal scans, such a signature results in a circular band of lower values which is thinner than the bright band. Criteria to identify the melting layer have been incorporated into operational products that are provided by some radar networks [38]. Such products fail in some situations and become less accurate at an increasing distance from the radar. When radar data are not sufficient to determine melting layer characteristics, melting layer estimates are obtained from models or other inputs.

Although based on empirical observations, there is extensive literature [30,39,40,41,42] that links the actual rain height with the altitude of the 0 °C isotherm. According to ITU-R P.839-4 model [43], the mean annual rain height above mean sea level ($h_{R}^{m}$) is obtained from the mean annual height of the 0 °C isotherm ($h_{0}^{m}$) as

(3) $$h_{R}^{m}=h_{0}^{m}+0.36\;\mathrm{km}.$$

For the direct model presented in this paper, we instead resort to the more accurate instantaneous rain height $h_{R}$, which is calculated from the instantaneous height of the 0 °C isotherm $h_{0}$ as

(4) $$h_{R}=h_{0}-\Delta h,$$

where Δh is an empirical parameter [44,45,46], whose value depends on many different variables. In the remainder of the paper we have set Δh=400 m. Clearly, being based upon current height values, (4) reveals more accurate than (3) which instead relies on just a fixed annual average.

In the perspective of a practical implementation, the instantaneous value of $h_{0}$, needed for the estimation of the rain rate can be obtained in two different manners. The first approach is to extract the information relevant to a short-term forecast for the 0 °C isotherm height from the numerical weather prediction (NWP) weather research and forecasting (WRF) model data, which are obtained by mixing either real data (observations and analyses) with idealized atmospheric conditions [47]. Alternatively, we can resort to the modern-era retrospective analysis for research and applications (MERRA) [48] global re-analysis database that provides, over very long periods, modelled data relevant to the 0 °C isotherm height. Since the 0 °C isotherm height shows a very strong periodicity over seasons, averaged data may provide sufficient approximation for the daily values [39].

**Figure 7:**
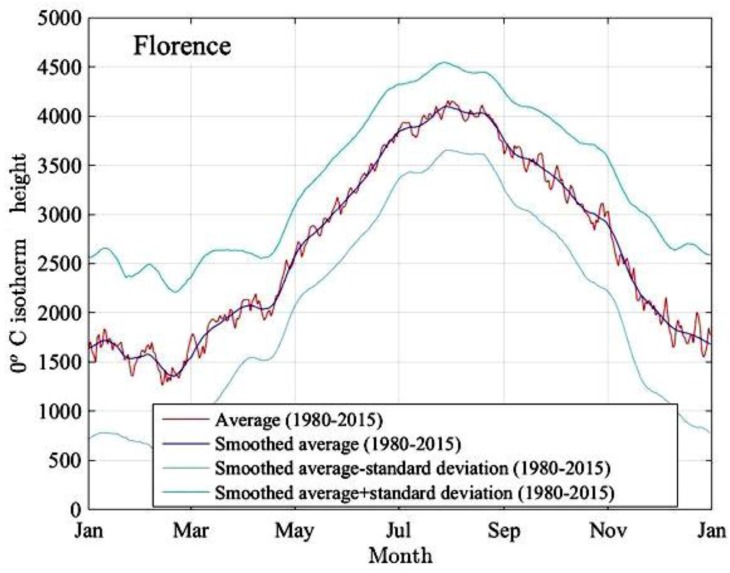
Daily height of the 0 °C isotherm over the city of Florence, Italy, obtained by averaging 35-year observation data from the modern-era retrospective analysis for research and applications (MERRA) database [48].

For example, Figure 7 shows, for each day of the year, the average value over 35 consecutive years of the 0 °C isotherm height over the city of Florence, Italy, obtained from the MERRA database. The red curve shows the raw data, the violet one plots the 0 °C isotherm height after some smoothing (i.e., low-pass data filtering), while the upper and lower curves are obtained by taking the smoothed average plus or minus one standard deviation of the measured data.

### 4.4. Experimental Derivation of the Analytical Regression Formula

More than 6 years of DSDs collected each minute by using a Parsivel disdrometer made by OTT Hydromet Deutschland (85,207 samples) in the area of Rome (the disdrometer is installed on the roof of the Institute of Atmospheric Sciences and Climate of the National Research Council, CNR) have been used as input of the T-matrix electromagnetic simulation method to compute the specific attenuation, according to the integral in (1), at different frequencies and for different polarizations. The used dataset is one of the longest time-series of measured DSD in Italy and include a huge amount of different types of precipitation events. The choice of using relationships derived by DSDs, instead of others available from the literature (in particular, those obtainable from the ITU-R P.838-3 recommendation [29]), is supposed to produce *R*-*k* relations that are more representative of the climate of the area in which the disdrometer measurements are collected. Since such a database is unique in Italy, it is not currently possible to quantify at what extent such relationships are representative for different regions. However, we can reasonably assume that they are valid at least for the climatic region in which Rome is included, which actually include almost all the regions of West Italy, like Tuscany [49,50]. The following assumptions have been considered in the T-matrix method: The environmental temperature of 20 °C [51]; fall velocity has been applied to compute *N*(*D*); drop shapes have been modelled with the [52] relationship; and the variability of the canting angle has been modelled with a Gaussian function with zero mean and 10° standard deviation. Furthermore, in the simulation, an elevation angle of 40° was assumed. The values of the rain rate can be straightforwardly obtained from measured DSDs. Once the values of *k* (in dB/km) and *R* (in mm/h) are known for a given DSD, the coefficients *α* and *β* of the following relation have been determined by performing a nonlinear regression

(5) $$R=\alpha \;k^{\beta }.$$

**Figure 8:**
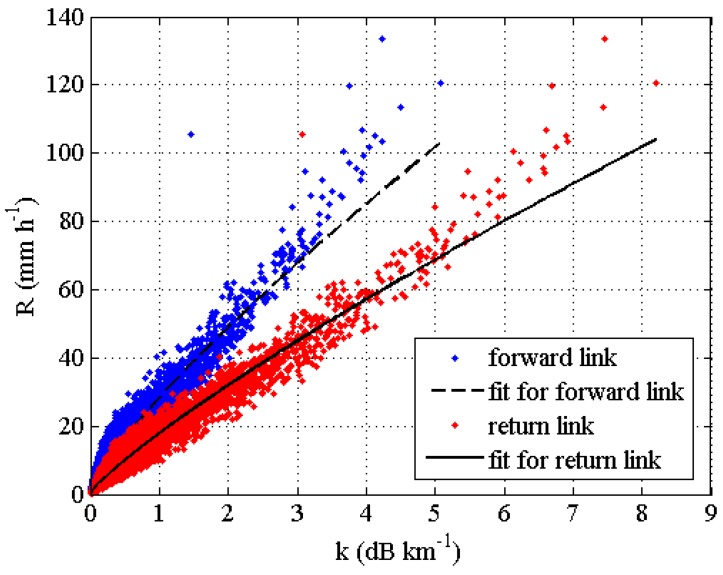
Scatterplot between rain rate *R* (in mm/h) and specific attenuation *k* (in dB/km) for the forward link (blue dots) and for the return link (red dots), both in the Ku-band. The two black lines represent the nonlinear regression of the data.

Figure 8 shows the regression fits between *R* and *k* for the forward link (i.e., 11,345.6 MHz with vertical polarization) and the return link (i.e., 14,216.6 MHz with horizontal polarization), both in Ku-band, but with different polarizations. Finally, Table 2 shows the values of the obtained parameters and those relative to the statistics computed, for both the polarizations, between disdrometer-based rain rate and the rain rate estimates through the *R-k* algorithms, namely the normalized mean absolute error (NMAE), normalized bias (NB), root mean square error (RMSE), and correlation coefficient (CC). NB is an index of the systematic error: Negative values means that the *R*-*k* relation slightly underestimates the rainfall rate with respect to disdrometer measured values. This statement is particularly valid for high rain rates (*R* > 80 mm/h). RMSE is a measure of the accuracy of the proposed algorithm; the obtained low values indicate an overall good performance of the *R*-*k* relation. NMAE values indicate the possible influence of the DSD variability on the accuracy of the rain rate estimates. The values of 17.9% and 20.6% indicates a limited impact of DSD variability. Figure 8 and the statistics mentioned above point out a fundamental advantage of choosing experimentally-derived relationships. In fact, in this way, we have a way to assess the influence of DSD variability on the selected relationship, which will be essential information when the performance of the NEFOCAST system will be characterized with respect to the different sources of error.

### 4.5. Implementation of the Direct Method

The direct method utilizes (5) with coefficients given by Table 2 and is estimated by means of nonlinear regressions applied to a large quantity of experimental data [12]. These coefficients are optimized for the specific climatic area where the experiment takes place (i.e., central Italy) and depend on electromagnetic parameters such as frequency and polarization. The method also relies on the knowledge of rain height, that can be evaluated as in (4), where the 0 °C isotherm height is obtained, e.g., from WRF short-term forecasts, and is adequately reduced by Δh∈(400 m,800 m). More specifically, the method envisages the following steps: (i) the SmartLNB measures the (negative) variation of $E_{s}/N_{0}$ (see Section 4.6), due to the presence of rain, with respect to clear-sky conditions; (ii) this measurement is then used to calculate the downlink rain attenuation $\hat{A}$; (iii) the length *L* of the slant path affected by rain is calculated by exploiting the knowledge of rain height and link geometry; (iv) the specific attenuation *k* is evaluated as $k=\hat{A}/L$; (v) the rainfall rate is eventually estimated from (5) as (see Section 4.4)

(6) $$\hat{R}=\alpha \;{\left(\hat{A}/L\right)}^{\beta }=\Psi (\hat{A})$$

Further details are provided in the next section. A variant of the foregoing procedure consists of replacing the analytical relationship (5) with experimental curves or nomograms available in the literature [53], which provide specific attenuation as a function of frequency, rainfall rate and polarization. An example of such curves is shown in Figure 5. Once the transmission frequency is chosen, the cited curves/nomograms provide a sampled version of the specific attenuation vs. rainfall rate, and only some preliminary interpolation between points is needed to carry out step (v) of the method.

### 4.6. Rain Rate Estimation Algorithm

The NEFOCAST rain rate estimation algorithm relies on the availability of the instantaneous value of the ratio $\eta =E_{s}/N_{0}$ between the received energy-per-symbol $E_{s}$ and the one-sided power spectral density of the additive white Gaussian noise $N_{0}$. The latter is the sum of the noise picked up by the receive antenna (expressed by the noise temperature $T_{A}^{}$) and the noise originated from the receiver electronics (represented by the noise temperature $T_{RX}^{}$). The presence of rain induces a specific additional path attenuation A (with A>1) which has a twofold effect: (i) it attenuates all RF waveforms (both the useful signal and the cosmic noise) coming from the outer space; (ii) it increases the atmospheric noise picked up by the receive antenna [54]. More specifically, denoting as $E_{s}{}^{\left(Clear\;Sky\right)}$ the energy $E_{s}$ received in “clear sky” conditions, i.e., in the absence of rain (A=1), *η* can be expressed as

(7) $$\eta =\frac{\frac{E_{s}{}^{\left(Clear\;Sky\right)}}{A}}{k_{B}\left[\underset{T_{A}}{\underset{︸}{\frac{T_{C}}{A_{atm}\cdot A}+T_{m}\left(1-\frac{1}{A_{atm}\cdot A}\right)+T_{G}}}+T_{RX}\right]}$$

where the meaning of parameters is specified in Table 3 along with their values. In detail, *T_m_* is the mean thermodynamic temperature of the meteorological formation, such as clouds and rain, which constitute an absorbent, and consequently emissive, medium (see [54], page 184). According to [55,56], a value of 275 K can be assumed for *T_m_*; however, we also numerically verified that taking values in the range 260–280 K (as formerly specified in [57]) has no appreciable effects on the values of the rain rate estimates. T_C_ and T_G_ are the noise temperatures of the cosmos and of the ground, respectively [54]), while *T_RX_* is the noise temperature of a commercial-grade receive system whose noise figure, referred to 290 K, is 0.2 dB. Finally, *A_atm_* is the gaseous attenuation of the atmosphere [54], which is given by the zenith attenuation for the specified link frequency (available from graphs) divided by the sine of the elevation angle of the satellite above the horizon.

After some manipulation, we get

(8) $$A=\frac{\left[\frac{T_{C}}{A_{atm}}+T_{m}\left(1-\frac{1}{A_{atm}}\right)+T_{G}+T_{RX}\right]\cdot \frac{\eta^{\left(Clear\;Sky\right)}}{\eta }+\frac{T_{m}-T_{C}}{A_{atm}}}{T_{m}+T_{G}+T_{RX}}=\Xi (\eta )$$

where $\eta^{\left(Clear\;Sky\right)}$ is the clear-sky value of *η*, which can be obtained letting A=1 in (7). Also, we notice that $\Xi (\eta^{\left(Clear\;Sky\right)})=1$. By using (8), the instantaneous value of *η* inferred from the received signal (denoted as $\hat{\eta }$) can be mapped into an estimate of the attenuation $\hat{A}$, i.e., $\hat{A}=\Xi (\hat{\eta })$.

Let us now assume a link geometry as depicted in Figure 6a, wherein the rain height *h_B_* is obtained either from a short-term forecast via WRF model or from an archive of historical data such as the MERRA. Thus, an estimate $\hat{k}$ of the specific attenuation (in dB/km) is derived under the assumption of uniform rain rate along the whole length of slant path affected by rain, so that $L=l^{\left(r\right)}=h_{B}/\sin\gamma$ and the estimate of the rain rate is obtained from (6) as

(9) $$R=\alpha \;{\left(\Xi (\hat{\eta })/l^{\left(r\right)}\right)}^{\beta }.$$

The performance of the NEFOCAST system will be affected by the variability of precipitation in space. Several authors have studied the variability of rainfall and its effects on the ground based or satellite-based remote sensing of precipitation (we limit ourselves to mention [58] for horizontal variability, [15,59] for vertical variability, the latter from the point of view of the impact on radar retrievals). The variability is related to the precipitation process. It is expected that, for stratiform rain, the variability of rainfall in time and space will be much lower than for convective rain. In our case, variability will affect: (1) the representativeness of the estimate of rainfall at ground from path-integrated measurements, and (2) the spatial density of LNB required to achieve a meaningful description of rainfall fields.

## 5. Experimental Results

The rain rate sensing capability of NEFOCAST ground terminals featuring the SmartLNB device was assessed experimentally during spring 2017. Tests were carried out by monitoring a Ku-band downlink signal from EUTELSAT 10A satellite using a ground station identified as NEFOCAST-ITA-PI-003-X, which is located at the Department of Information Engineering of the University of Pisa, Italy. The main parameters and features of the satellite link are listed in Table 4. The SmartLNB continuously takes samples of the received signal and uses an embedded proprietary algorithm to evaluate the instantaneous $\eta =E_{s}/N_{0}$, with 0.1 dB resolution. The update rate can be set by the user. During the experimenta­tion, the samples of the received signal were processed every 20 s to measure the rain-induced attenuation affecting the received signal. Experimental calibration of the receiver in clear-sky conditions yielded $\eta^{\left(Clear\;Sky\right)}=11.4\;\mathrm{dB}$. The rain height $h_{R}$ was calculated as in (4), where the 0 °C isotherm height $h_{0}$ was given by short-term forecasts issued by LaMMA Consortium (as the regional operational meteorological service of Tuscany Region) and the offset was set at Δh=400 m. Parameters *α* and *β* of the *R*-*k* algorithm were taken from Table 2, for the case of vertical polarization (forward link). These rain rate estimates, ultimately derived from the measurements of $E_{s}/N_{0}$, i.e., of satellite signal quality, were eventually compared with the data provided by a conventional tipping-bucket rain gauge Davis PRO2 (0.2 mm resolution) co-located with the SmartLNB receiver.

**Figure 9:**
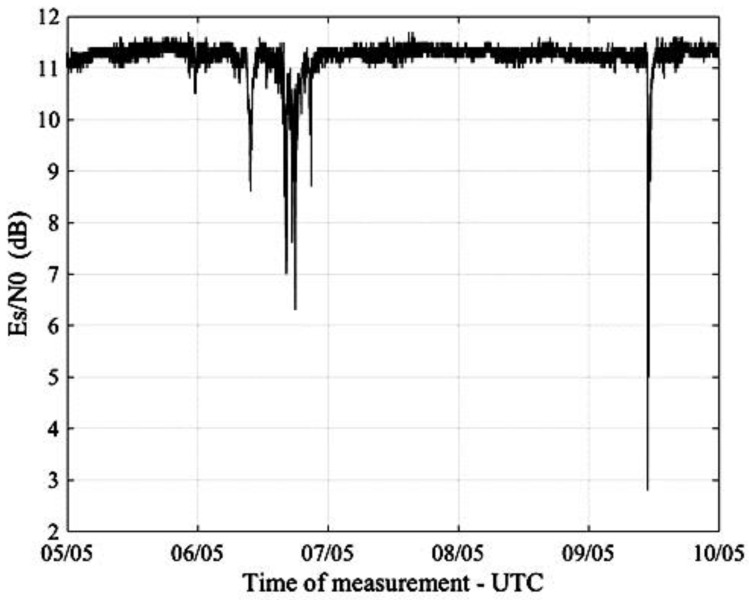
$E_{s}/N_{0}$ provided by the SmartLNB in Pisa, Italy, 5–10 May 2017.

**Figure 10:**
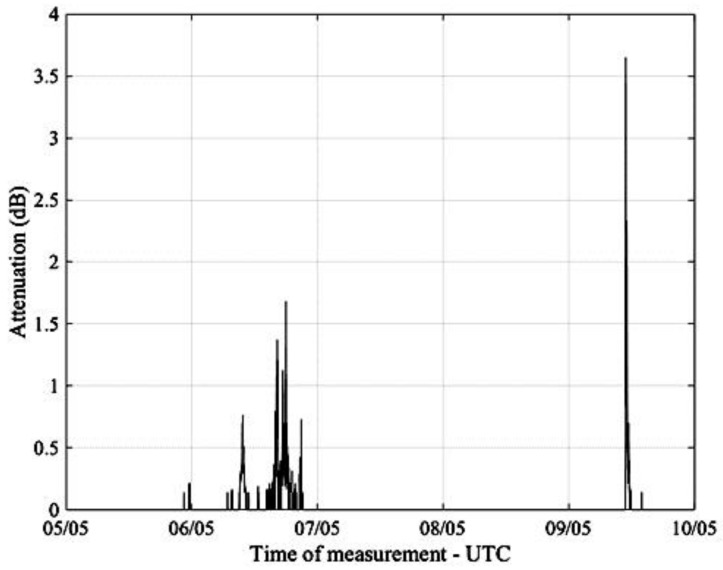
Calculated signal attenuation in Pisa, Italy, 5–10 May 2017.

Figure 9 shows the plot of a real-time measurement of $E_{s}/N_{0}$ obtained from a SmartLNB located in Pisa, Italy, 5–10 May 2017, while Figure 10 presents the corresponding signal attenuation, calculated from (8).

**Figure 11:**
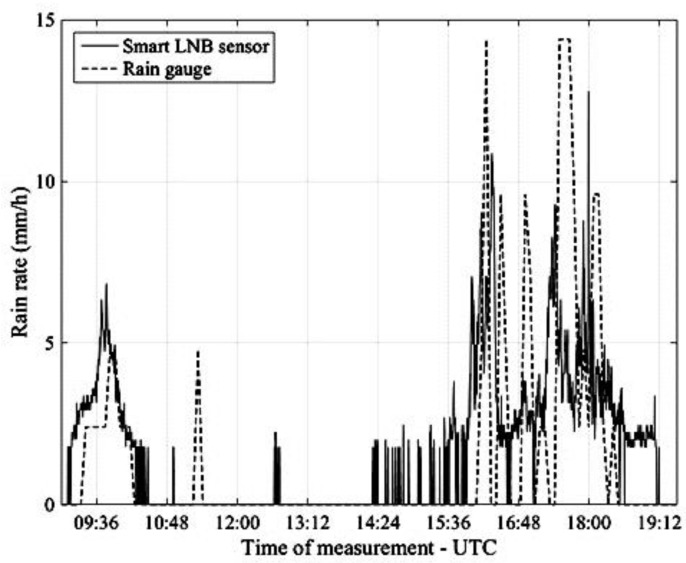
Pisa, 6 May 2017. Rain rate estimates provided by the NEFOCAST SmartLNB-based sensor (solid line) and by the TBR (dashed line).

We now focus on a few significant rain events which occurred in Pisa on 6 May and 9 May 2017. On both days, LaMMA’s short term forecasts indicated that the 0 °C isotherm height was $h_{0}≅2.4\;\mathrm{km}$. Figure 11 is relative to the rain event which occurred on 6 May and compares the rain rate estimated every 20 s by the NEFOCAST system (solid line) with the estimates provided every 5 min by the TBR (dashed line), according to the following formula

(10) $$\hat{R}=\left(\frac{n_{tips}\cdot {\Delta |}_{\mathrm{mm}}}{5\;\min}\right)\cdot 60\;\frac{\min}{h},$$

where ${\Delta |}_{\mathrm{mm}}=0.2\;\mathrm{mm}$ is the tipping resolution, i.e., the measured rainfall per tip, and $n_{tips}$ is the number of tips counted in a 5-minute interval.

**Figure 12:**
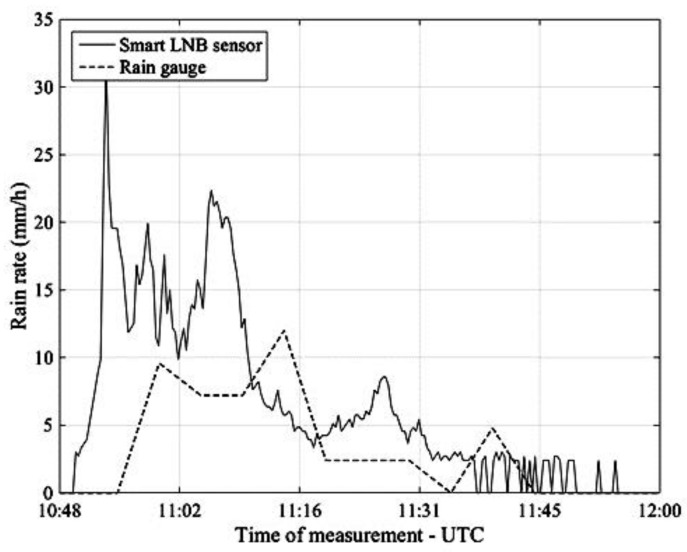
Pisa, 9 May 2017. Rain rate estimates provided by the NEFOCAST SmartLNB-based sensor (solid line) and by the TBR (dashed line).

Figure 12 refers to the rain event occurred in Pisa on 9 May and as with the previous figure (albeit with a different scale) compares NEFOCAST real-time rain rate estimates (solid line) with the quantized estimates provided by the TBR (dashed line).

**Figure 13:**
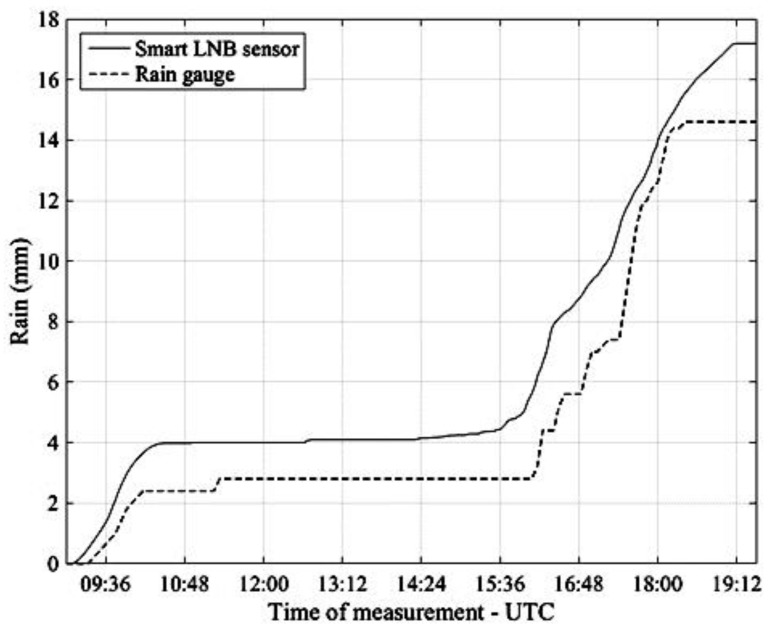
Pisa, 6 May 2017. Accumulated rainfall estimated by the NEFOCAST SmartLNB sensor (solid line) and measured by the TBR (dashed line).

Figure 13 refers to the rain event which occurred in Pisa on 6 May 2017 and compares the accumulated rainfall calculated by integrating the NEFOCAST rain rate estimates (solid line) with the measurements provided by the TBR (dashed line).

**Figure 14:**
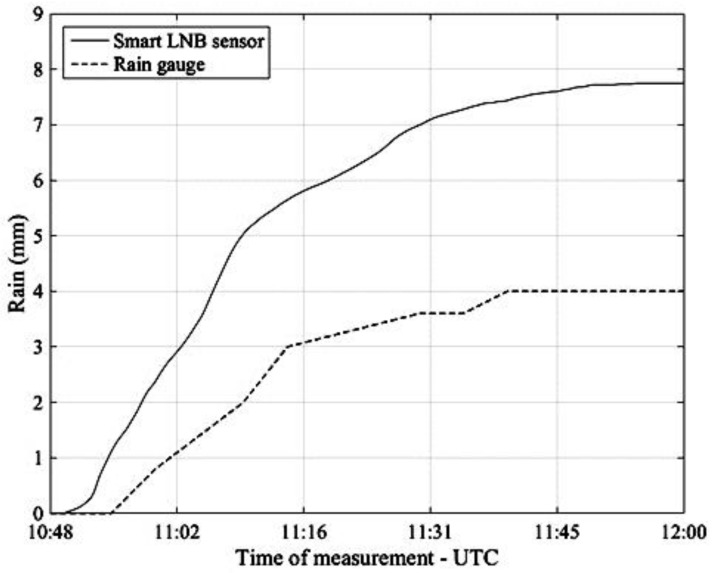
Pisa, 9 May 2017. Accumulated rainfall estimated by the NEFOCAST SmartLNB sensor (solid line) and measured by the TBR (dashed line).

Finally, Figure 14 refers to the rain event of 9 May 2017 and, as in the previous Figure 13, compares the accumulated rainfall obtained by integration of the NEFOCAST rain rate estimates (solid line) with the measurements provided by the rain gauge (dashed line).

An inspection of Figure 11, Figure 12, Figure 13 and Figure 14 reveals that, thanks to the very short (20 s) update interval, the NEFOCAST sensor is capable of monitoring and following accurately the fast and random evolution of the rainfall rate during a precipitating event, and warrants a much faster response than a conventional tipping bucket gauge. Furthermore, the latter seems less reliable at medium-to-low rainfall rates, due to its quantized response (10) and resolution, that do not allow the determination of exactly when precipitation starts and when it presents spikes. On the other hand, the NEFOCAST sensor seems less reliable in estimating the cumulated rainfall over long periods, since the attenuation measured by the SmartLNB depends not only on the instantaneous rain rate falling in the installation site, but also on the rain distribution over the entire length of the slant path affected by the rain. This may introduce a nonnegligible random bias when integrating the instantaneous estimated rain rate over long periods of time.

In this respect, it is worth recalling that the proposed rainfall estimation technique is affected by many sources of error, a few of which were mentioned earlier. They can be roughly classified as (i) errors in the *R*-*k* mapping law, i.e., in the choice of the coefficients involved in (5), notably when they are derived by means of global models such as [29]; (ii) errors due to the (unknown) variability of the rainfall rate along the electromagnetic path, resulting in an (averaged) specific attenuation measurement that does not exactly match the desired local specific attenuation at the SmartLNB site; (iii) errors in estimating the liquid precipitation height and other geometric parameters; (iv) errors in the knowledge of those parameters related to the propagation medium and the environment surrounding the receiver, such as climatic conditions, ground temperature and the like. A criterion pursued in the NEFOCAST project and aimed at reducing the impact of the above inaccuracies was to use, wherever possible, measured or reliably estimated (e.g., by short-term forecasts) coefficients and parameters, avoiding the use of global standardized statistical models. The latter are indeed very useful tools to easily obtain predictions valid anywhere in the world but, for a specific site, a better accuracy can be attained from local experimental data. As mentioned earlier, we adhered to this criterion in Section 5 for the determination of the coefficients in (5) and the 0 °C isotherm height.

## 6. Conclusions and Future Work

In the framework of the NEFOCAST project, we have investigated the feasibility of using a network of new-generation Eutelsat SmartLNB interactive satellite terminals for widespread and capillary monitoring of precipitation fields throughout the Tuscan Region. In addition to operating as a terminal for interactive domestic DVB-S services in the Ku and Ka bands, each of these devices can serve also as a weather sensor. They have a fine capability for measuring rain-induced instantaneous attenuation on the signal received from the forward downlink and relaying these attenuation data over a narrowband satellite return link to the NEFOCAST service center, gathering real-time information from all the SmartLNBs active in the territory. The service center processes the collected attenuation data to reconstruct, and thus monitor, the precipitation field on the territory, also presenting the ability to alert civil protection services in the presence of potentially critical meteorological situations. In particular, we discussed some methods to convert the signal attenuation measurements into corresponding instantaneous rainfall rates. The most reliable among the assessed techniques is based on the use of an exponential model describing the dependence between the specific rain attenuation *k* (dB/km) and the rainfall rate *R* (mm/h); the coefficients in the model being related to the specific geographic zone, to the operating frequency and to the signal polarization. Numeric values for these coefficients were numerically obtained by fitting the above exponential law with extensive experimental data. The algorithm computes the specific signal attenuation starting from the total rain-induced attenuation provided by the NEFOCAST terminal, using a few geometric link data and the rain field height. This latter can be derived either from short-term forecasts of the 0 °C isotherm height provided by a local area model, or (albeit with some accuracy loss) it can be replaced by a statistical estimate based on long-term observations. Some preliminary results obtained from a SmartLNB operating in Pisa during precipitation events occurred in May 2017 were presented and compared with the readings of a conventional TBR. The results indicate that the NEFOCAST sensor, thanks to its short sampling interval (of the order of a few seconds), is able to track and monitor accurately the fast and random evolution of the rainfall rate during a precipitating event and has a much faster response than a conventional tipping bucket gauge.

Subsequent refinements of the algorithms should be implemented to reduce some source of the errors; for example, the introduction of ancillary data (i.e., satellite data) for discriminating stratiform and convective systems could improve the results.

Although these are preliminary outcomes obtained for a single terminal in the experimental area, the results are encouraging for the future activities of NEFOCAST, which aims to set up a scaled demonstrator of the NEFOCAST network, including the service center, with a sufficiently wide set of terminals to cover a significant portion of the territory. This network will be devoted not only detect, monitor and map the instantaneous precipitation field, but also to nowcasting applications regarding surveillance and alerting activities in case of potentially dangerous meteorological events.

## Figures and Tables

**Table 1:** NEFOCAST Space Segment.

	Forward Link	Return Link
Satellite	EUTELSAT 10A	EUTELSAT 10A
Frequency	11,345.8 MHz	14,216.6 MHz
Polarization	Vertical	Horizontal
Physical Layer Protocol	DVB-S2	F-SIM
Link Layer Protocol	IP-MPE	F-SIM
EIRP	48 dBW	-
G/T	-	+4 dB/K
MODCOD	QPSK 4/5	-
Spreading Factor	-	16, 32, 64, 128, 256

**Table 2:** Parameters *α* and *β* of the *R-k* algorithms and statistics indices between measured and estimated rain rates, in the Ku-band.

*R* = *α k^β^*	*α*	*β*	NMAE	NB	RMSE	CC
Return link 14,216.6 MHz, H polarization.	17.910	0.836	17.9%	−4.3%	0.90	0.983
Forward link 11,345.5 MHz, V polarization.	28.140	0.798	20.6%	−4.4%	1.07	0.976

**Table 3:** Parameters for the evaluation of $E_{s}/N_{0}$.

Parameter	Value
*k*_B_: Boltzmann constant	1.38 × 10^−23^ J/K
*T_C_*: Cosmic noise temperature	2.78 K
*T_G_*: Ground noise temperature	50 K
*T_m_*: Mean thermodynamic temp. of the atm. formations	265 K
*T_RX_*: Receiver noise temperature	13.67 K
*A_atm_*: Gaseous atmospheric attenuation	0.13 dB

**Table 4:** Features of the satellite link used to test the rain sensing system.

Parameter/Feature	Value/Description
Satellite	EUTELSAT 10A
Longitude	10° East
Elevation angle	39.6°
EIRP	48 dBW
Transponder Bandwidth	36 MHz
Frequency	11,345.833 MHz
Polarization	Vertical
Receiver Antenna Diameter	75 cm
LNB Noise Figure @ 290 K	0.2 dB
Physical Layer Protocol	DVB-S2
Symbol Rate	29.00 MBaud
MODCOD	QPSK 4/5
Ground Station Coordinates	43.72031° North 10.38377° East
